# Association Between Ischemic Stroke and Tumor Necrosis Factor Inhibitor Therapy in Patients With Rheumatoid Arthritis

**DOI:** 10.1002/art.39582

**Published:** 2016-05-26

**Authors:** Audrey S. L. Low, Mark Lunt, Louise K. Mercer, Kath D. Watson, William G. Dixon, Deborah P. M. Symmons, Kimme L. Hyrich

**Affiliations:** ^1^ Arthritis Research UK Centre for Epidemiology, Centre for Musculoskeletal Research, Institute of Inflammation and Repair, The University of Manchester and Salford Royal Hospital NHS Foundation Trust; ^2^ Arthritis Research UK Centre for Epidemiology, Centre for Musculoskeletal Research, Institute of Inflammation and Repair, The University of Manchester; ^3^ Arthritis Research UK Centre for Epidemiology, Centre for Musculoskeletal Research, Institute of Inflammation and Repair, The University of Manchester and NIHR Manchester Musculoskeletal Biomedical Research Unit, Central Manchester NHS Foundation Trust, Manchester Academic Health Science Centre, and Salford Royal Hospital NHS Foundation Trust; ^4^ Arthritis Research UK Centre for Epidemiology, Centre for Musculoskeletal Research, Institute of Inflammation and Repair, The University of Manchester and NIHR Manchester Musculoskeletal Biomedical Research Unit, Central Manchester NHS Foundation Trust, Manchester Academic Health Science Centre, Manchester, UK

## Abstract

**Objective:**

Patients with rheumatoid arthritis (RA) are at an increased risk of ischemic stroke. Tumor necrosis factor inhibitors (TNFi) may influence risk and mortality after ischemic stroke by reducing inflammation. This study was undertaken to examine the association of TNFi with the risk of incident ischemic stroke and with 30‐day and 1‐year mortality after ischemic stroke.

**Methods:**

Patients with RA starting therapy with TNFi and a biologics‐naive comparator group treated with synthetic disease‐modifying antirheumatic drugs (DMARDs) only were recruited to the British Society for Rheumatology Biologics Register for Rheumatoid Arthritis from 2001 to 2009. Patients were followed up via clinical and patient questionnaires as well as the national death register. Incident strokes were classified as ischemic if brain imaging reports suggested ischemia or if ischemic stroke was reported as the underlying cause of death on a death certificate. Patients with a previous stroke were excluded. Risk of ischemic stroke was compared between patients receiving synthetic DMARDs only and those ever‐exposed to TNFi using a Cox proportional hazards regression model adjusted for potential confounders. Mortality after ischemic stroke was compared between synthetic DMARD–treated patients and TNFi‐treated patients using logistic regression, adjusted for age and sex.

**Results:**

To April 2010, 127 verified incident ischemic strokes (21 in 3,271 synthetic DMARD–treated patients and 106 in 11,642 TNFi‐treated patients) occurred during 11,973 and 61,226 person‐years of observation, respectively (incidence rate 175 versus 173 per 100,000 person‐years). After adjustment for confounders, there was no association between ever‐exposure to TNFi and ischemic stroke (hazard ratio 0.99 [95% confidence interval (95% CI) 0.54–1.81]). Mortality 30 days or 1 year after ischemic stroke was not associated with concurrent TNFi exposure (odds ratio 0.18 [95% CI 0.03–1.21] and 0.60 [95% CI 0.16–2.28], respectively).

**Conclusion:**

Exposure to TNFi does not appear to influence the occurrence of ischemic stroke in the medium term in patients with RA. The impact on mortality after ischemic stroke remains inconclusive.

Patients with rheumatoid arthritis (RA) are at increased risk of cardiovascular morbidity and mortality, especially myocardial infarction (MI), compared to healthy subjects 1, 2. The epidemiology of stroke in RA is not as well studied as that of MI, with some studies demonstrating an increased risk of stroke 3, 4, 5, 6 and others finding no association between RA and stroke 7, 8, 9, 10. Strokes can be classified into 2 subtypes: ischemic and hemorrhagic. In the majority of previous studies, these subtypes were analyzed as a composite event, which may account for the differing results observed. However, in an analysis of the association of ischemic stroke with RA, the authors reported an almost 3‐fold increase in risk in the US National Databank of Rheumatic Diseases (NDB) 5. A somewhat lower association was observed in a Swedish study, but the risk still appeared to be increased in patients with RA (relative risk 1.1–1.2) 6.

Inflammation has been proposed as a potential mediator of the atherosclerotic process leading to ischemic stroke, with tumor necrosis factor (TNF) as one of the key drivers of this process 11. Control of inflammation through blockade of TNF may lead to a reduction in the risk of ischemic stroke. In the NDB, there was no association between the risk of ischemic stroke and exposure to TNF inhibitors (TNFi) after adjustment for disease severity factors, low‐dose aspirin use, and comorbidity 5. In an analysis of Medicare records, Solomon et al found no significant association of stroke (all subtypes) with TNFi compared to methotrexate (MTX) monotherapy 12. These studies were conducted over a relatively short follow‐up period of 1–2 years, whereas any influence of exposure to TNFi on the risk of ischemic stroke may occur over a longer period.

TNF may also play a role in influencing outcome after a stroke. In animal models of ischemic stroke, antibodies to TNF have been found to reduce the postischemic infarct volume of the brain and protect neuronal cells against further ischemic damage 11. Conversely, TNF itself also regulates the tolerance of the brain to hypoxia and ischemia after a stroke, so blockade of TNF may be harmful to a stroke patient 13.

The aims of this analysis were to compare (a) the occurrence of incident ischemic stroke and (b) the 30‐day and 1‐year all‐cause mortality following ischemic stroke in a cohort of patients with RA receiving TNFi therapy with that in a comparator cohort of biologics‐naive patients with RA receiving synthetic disease‐modifying antirheumatic drug (DMARD) therapy.

## PATIENTS AND METHODS

### Participants and study design

Patients included in this study were participants in the British Society for Rheumatology Biologics Register for Rheumatoid Arthritis (BSRBR‐RA) 14. The BSRBR‐RA is a national prospective observational cohort study of patients starting treatment with TNFi and other biologic agents for RA that has been undertaken in order to examine long‐term safety.

UK guidelines restrict the prescription of TNFi therapy to subjects with RA with active disease (Disease Activity Score in 28 joints [DAS28] [15] >5.1 measured on 2 occasions at least a month apart, and have undergone trials of 2 synthetic DMARDs, including MTX, unless contraindicated) 16. Patients in the TNFi‐treated cohort included in this analysis were biologics naive at baseline and had to be registered within 6 months of starting adalimumab, etanercept, or infliximab, to minimize left censorship. A biologics‐naive comparator cohort of patients with active RA (guide DAS28 ≥4.2) receiving synthetic DMARD therapy only was also recruited between 2001 and 2009. If patients in the synthetic DMARD comparator cohort were switched to biologic therapy because of severe disease, they were given the option to reconsent to be recruited to the biologics cohort if recruitment to that particular drug cohort was still open; otherwise, follow‐up was stopped after the patient started taking a biologic drug.

Both cohorts were recruited and followed up using identical study questionnaires sent to both the patient's rheumatology team and the patient. Data captured from the rheumatology team included disease details, medication, and comorbidity, as well as the occurrence of adverse events. Patients also provided information on hospitalizations. In addition, all patients were flagged for death reporting with the Health and Social Care Information Centre. In the event of a death, a copy of the death certificate was sent to BSRBR‐RA, with causes of death coded using the International Statistical Classification of Diseases and Related Health Problems, Tenth Revision (ICD‐10). For all reports of a serious adverse event, additional clinical data (e.g., supporting clinical details, discharge summaries, and pathology, radiology, and laboratory reports) were requested from the treating rheumatologist to aid event verification and classification.

In order to be included in the analysis, patients had to have at least one clinical questionnaire returned to the study coordination center. The analysis was also restricted to patients who had at least moderate disease activity at baseline (DAS28 >3.2) 17. Patients with a prior stroke recorded at baseline were excluded. Written informed consent was obtained from all patients in accordance with the Declaration of Helsinki. Ethics approval for this study was obtained from the North West Multicentre Research Ethics Committee (reference no. MREC 00/8/53).

### Drug exposure model

The primary outcome for this analysis was the first verified ischemic stroke per patient. An ever‐exposed drug exposure model was chosen as the primary analysis model; patients were not censored if they switched to a non‐TNFi drug. The 3 TNFi drugs included in this analysis were analyzed as a single exposure group. Follow‐up was censored at first verified ischemic stroke, at death, on April 30, 2010, or on the date of the last returned follow‐up questionnaire, whichever came first. Sensitivity analyses included 1) analyzing the risk of first verified ischemic stroke with 2 different drug exposure models, (a) on TNFi and (b) on TNFi plus 90‐day lag window, and 2) the risk of all verified ischemic strokes over time from start of TNFi therapy.

### Identification, verification, and subtyping of strokes

All potential strokes reported via questionnaires to the BSRBR‐RA were verified using all clinical data supplied. These potential strokes were verified against the World Health Organization (WHO) criteria, which state that **“**a stroke is defined as a focal or global neurological impairment of sudden onset, lasting for more than 24 hours (or leading to death) and of presumed vascular aetiology**”** 18. Two clinicians (ASLL and LKM) independently verified each reported case of stroke, with any disagreement resolved by consensus. All cases were presumed to have a vascular etiology unless stated otherwise. Cases with no or scarce additional clinical information with which to verify the event were excluded from the analysis. Cases in which the only report of a stroke was on a death certificate were also accepted as a verified stroke if the underlying cause of death was coded as ICD‐10 I60‐I64.

All verified strokes were further classified into ischemic, hemorrhagic, or unclassifiable strokes. This was done in 2 ways. First, results of available computed tomography (CT) or magnetic resonance (MR) brain imaging reports were reviewed. Reports showing an infarct or reported as normal were classified as ischemia stroke; those showing a brain hemorrhage were classified as hemorrhagic strokes. Second, where a stroke was identified from a death certificate, the underlying cause of death was used to classify the stroke: ischemic (ICD‐10 code I63) or hemorrhagic (codes I60‐62). All verified strokes in patients who had no available CT or MR brain imaging reports or had an underlying cause of death of ICD‐10 code I64 (stroke, not specified as hemorrhage or infarction) were “unclassified.” The risk of all first verified strokes (hemorrhagic, ischemic, and unclassified subtypes) was analyzed in a sensitivity analysis.

### Statistical analysis

Crude incidence rates of ischemic stroke with 95% confidence intervals (95% CIs) were calculated assuming a Poisson distribution. The risk of first ischemic stroke was compared between the synthetic DMARD**–**treated patients and TNFi‐treated patients using a Cox proportional hazards regression model, adjusted for deciles of propensity scores. This was presented using hazard ratios (HRs) with 95% CIs. The variation in the risk of all ischemic strokes over time was also presented using HRs with 95% CIs.

Potential baseline confounders were specified a priori and entered into a logistic regression model to generate a propensity score, reflecting the likelihood of receiving the exposure of interest (in this case, TNFi) depending on covariates. These covariates were age, sex, DAS28, disease duration, Health Assessment Questionnaire score 19, whether the patient had ever used ≥4 synthetic DMARDs (yes/no), date of registration in the BSRBR‐RA (dichotomized as before or after June 30, 2004), hypertension, diabetes, previous angina/MI, chronic lung disease, smoking status, glucocorticoid use, nonsteroidal antiinflammatory drug (NSAID) and cyclooxygenase (COX) inhibitor use, antiplatelet therapy (aspirin, clopidogrel, and dipyridamole), statin use, and digoxin and/or warfarin use as a proxy for atrial fibrillation (AF). (AF was analyzed as a single variable.) Use of ≥4 synthetic DMARDs and date of registration were used as variables to account for unmeasured confounding relating to temporal changes in the way rheumatologists treated patients with RA. The balance of covariates between the exposure groups was examined by checking the expected bias after stratification by deciles of propensity score, since this method demonstrated the lowest degree of expected bias (see Supplementary Figure 1 and Supplementary Table 1, available on the *Arthritis & Rheumatology* web site at http://onlinelibrary.wiley.com/doi/10.1002/art.39582/abstract). Missing data were replaced using multiple imputation (Supplementary text, http://onlinelibrary.wiley.com/doi/10.1002/art.39582/abstract). The imputation model included whether the patient experienced a stroke (yes/no), logarithm of the time to ischemic stroke, and the other covariates described above.

Thirty‐day and 1‐year all‐cause mortality after ischemic stroke was compared between synthetic DMARD–treated patients and TNFi‐treated patients. All ischemic strokes identified only via death certificates were categorized as deaths within 30 days of a first ischemic stroke. All deaths after ischemic stroke occurring up to April 30, 2011 were included (i.e., 1 year after the last day of follow‐up). Three groups of patients were analyzed: 1) patients treated with synthetic DMARDs only, 2) patients receiving TNFi on or within 90 days of first missed dose, and 3) past exposure to TNFi at the time of ischemic stroke. All‐cause mortality after ischemic stroke was compared between patients treated with synthetic DMARDs and those treated with TNFi using logistic regression, adjusted for age and sex, and presented as odds ratios (ORs) with 95% CIs.

## RESULTS

### Baseline characteristics of the patients

**Figure 1:**
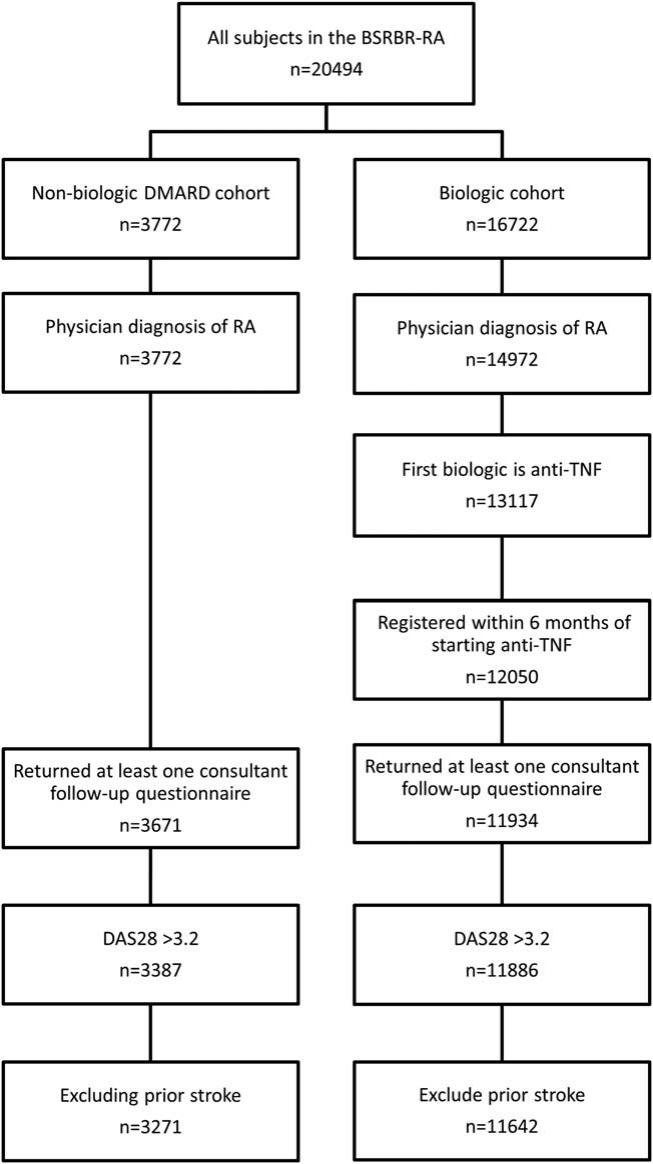
Patient selection for the analysis of ischemic stroke. BSRBR‐RA = British Society for Rheumatology Biologics Register for Rheumatoid Arthritis; DMARD = disease‐modifying antirheumatic drug; anti‐TNF = anti–tumor necrosis factor; DAS28 = Disease Activity Score in 28 joints.

A total of 14,913 patients (3,271 receiving synthetic DMARDs and 11,642 receiving TNFi) were included in the analysis (Figure 1). Compared to the synthetic DMARD**–**treated cohort, the TNFi‐treated cohort included younger patients, more women, and patients with longer disease duration and higher disease activity and functional disability at baseline (Table 1). Patients in the TNFi‐treated cohort were also more likely to be receiving glucocorticoids and antiinflammatory drugs (NSAIDs and/or COX‐2 inhibitors) but were less likely to be receiving antiplatelet drugs and statins and had a lower frequency of smoking, hypertension, and diabetes compared to patients in the synthetic DMARD**–**treated cohort at baseline. The median duration of TNFi therapy was 4.1 years (interquartile range 2.0**–**5.8).

**Table 1:** Baseline characteristics of synthetic DMARD–treated and TNFi‐treated patients^a^

	Patients receiving synthetic DMARDs (n = 3,271)	Patients receiving TNFi (n = 11,642)
Age, mean ± SD years	59.9 ± 12.3	56.0 ± 12.2
Sex, % female	73.5	76.5
DAS28, mean ± SD	5.3 ± 1.1	6.6 ± 1.0
Disease duration, median (IQR) years	6 (1–15)	11 (6–19)
HAQ score, mean ± SD	1.5 ± 0.7	2.0 ± 0.6
Patients exposed to ≥4 synthetic DMARDs, %	21.1	52.0
Recruited before June 30, 2004, %	19.2	51.7
Hypertension, %	31.2	29.5
Diabetes, %	6.6	5.6
Angina/MI, %	9.3	5.5
Chronic lung disease, %	19.2	13.5
Current/previous smoker, %	63.0	59.5
Glucocorticoid treatment, %	22.4	44.2
Antiinflammatory treatment (NSAIDs and/or COX‐2 inhibitors), %	55.3	62.7
Antiplatelet treatment, %	11.3	6.8
Statin treatment, %	12.8	7.1
Digoxin/warfarin treatment, %	2.1	1.7

a DMARD = disease‐modifying antirheumatic drug; TNFi = tumor necrosis factor inhibitor; DAS28 = Disease Activity Score in 28 joints; IQR = interquartile range; HAQ = Health Assessment Questionnaire; MI = myocardial infarction; NSAIDs = nonsteroidal antiinflammatory drugs; COX‐2 = cyclooxygenase 2.

### Risk of ischemic stroke and all stroke subtypes

**Figure 2:**
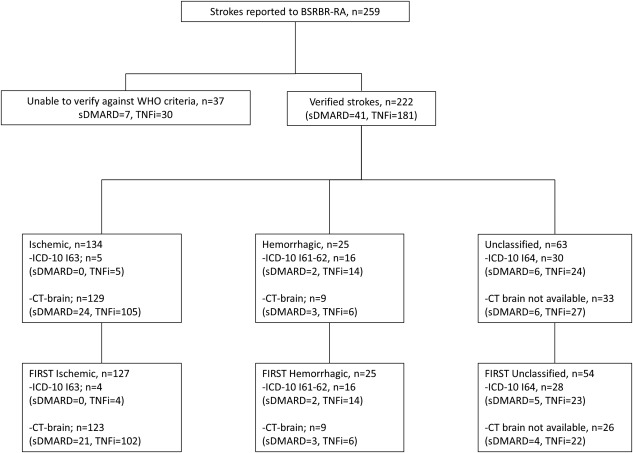
Stroke verification process. BSRBR‐RA = British Society for Rheumatology Biologics Register for Rheumatoid Arthritis; WHO = World Health Organization; sDMARD = synthetic disease‐modifying antirheumatic drug; TNFi = tumor necrosis factor inhibitor; ICD‐10 = International Statistical Classification of Diseases and Related Health Problems, Tenth Revision; CT = computed tomography.

There were 259 potential strokes reported to the BSRBR‐RA, of which 222 (86%) were verified (Figure 2). Of the 37 strokes that were unverifiable, 30 (81%) were in patients in the TNFi‐treated cohort. Of the 222 verified strokes, a similar proportion (82%; n = 181) was observed in the TNFi‐treated cohort, suggesting nondifferential verification between drug cohorts.

Of the 222 verified strokes, there were 134 ischemic, 25 hemorrhagic, and 63 unclassifiable strokes. The proportion of each stroke subtype was similar in the synthetic DMARD–treated and TNFi‐treated cohorts. Of the 222 verified strokes, 206 were first strokes (127 ischemic, 25 hemorrhagic, and 54 unclassifiable). Of the 127 first ischemic strokes, 21 occurred in the synthetic DMARD–treated cohort and 106 occurred in the TNFi‐treated cohort. The median duration of follow‐up per patient was 3.9 years in the synthetic DMARD–treated cohort and 5.6 years in the TNFi‐treated cohort.

Crude incidence rates of first ischemic stroke per 100,000 person‐years were 175 (95% CI 109–268) in the synthetic DMARD–treated cohort and 173 (95% CI 141–209) in the TNFi‐treated cohort (Table 2). Compared to the synthetic DMARD–treated patients, the unadjusted HR for risk of first ischemic stroke in TNFi‐treated patients was 1.04 (95% CI 0.65–1.66). After adjustment using deciles of propensity score, there was no significant association between the risk of first ischemic stroke and ever‐exposure to TNFi therapy (HR 0.99 [95% CI 0.54–1.81]) (Table 2). An analysis of all first strokes (35 in the synthetic DMARD–treated cohort and 171 in the TNFi‐treated cohort) also showed no association with ever‐exposure to TNFi therapy (adjusted HR 0.93 [95% CI 0.59–1.46]). Trimming of the cohort at the extremes of the propensity score did not affect these estimates.

**Table 2:** Risk of first stroke with exposure to TNFi therapy^a^

	Patients receiving synthetic DMARDs (n = 3,271)	Patients receiving TNFi (n = 11,642)
Years of follow‐up per patient, median (IQR)	3.9 (2.1–5.2)	5.6 (3.9–6.9)
Total person‐years of follow‐up	11,973	61,226
Ever‐exposure to TNFi, first ischemic stroke		
Number of verified first ischemic strokes	21	106
Crude incidence rate per 100,000 person‐years (95% CI)	175 (109–268)	173 (141–209)
Unadjusted HR (95% CI)	Referent	1.04 (0.65–1.66)
HR adjusted for age and sex (95% CI)	Referent	1.44 (0.89–2.32)
Fully adjusted HR stratified by deciles of propensity score (95% CI)	Referent	0.99 (0.54–1.81)
On‐drug, first ischemic stroke		
Number of verified first ischemic strokes	21	76
Unadjusted HR (95% CI)	Referent	0.93 (0.57–1.51)
HR adjusted for age and sex (95% CI)	Referent	1.37 (0.84–2.26)
Fully adjusted HR stratified by deciles (95% CI)	Referent	0.81 (0.43–1.55)
On‐drug + 90 days, first ischemic stroke		
Number of verified first ischemic strokes	21	88
Unadjusted HR (95% CI)	Referent	1.05 (0.65–1.69)
HR adjusted for age and sex (95% CI)	Referent	1.52 (0.94–2.48)
Fully adjusted HR stratified by deciles (95% CI)	Referent	1.03 (0.55–1.91)
Ever‐exposure to TNFi, all first strokes		
Number of verified first strokes (all subtypes)	35	171
Crude incidence rate per 100,000 person‐years (95% CI)	293 (204, 407)	280 (239–325)
Unadjusted HR (95% CI)	Referent	1.01 (0.70–1.46)
HR adjusted for age and sex (95% CI)	Referent	1.46 (1.00–2.12)
Fully adjusted HR stratified by deciles of propensity score (95% CI)	Referent	0.93 (0.59–1.46)

a TNFi = tumor necrosis factor inhibitor; DMARDs = disease‐modifying antirheumatic drugs; IQR = interquartile range; 95% CI = 95% confidence interval; HR = hazard ratio.

Other drug exposure models were explored in sensitivity analyses. Compared to patients receiving synthetic DMARDs, the HR for the risk of first ischemic stroke in patients receiving TNFi (on‐drug model) was 0.81 (95% CI 0.43–1.55), while the HR for the risk of first ischemic stroke in patients on TNFi plus lag period of 90 days was 1.03 (95% CI 0.55–1.91) (Table 2).

### Variation in risk of ischemic stroke over time

The absolute risk of ischemic stroke was low in both groups, and there were no ischemic stroke events in the synthetic DMARD**–**treated cohort in the fourth year of follow‐up, so it was not possible to obtain a comparison for each year of follow‐up (unable to divide by 0). All ischemic stroke events were included in this analysis (24 in the cohort receiving synthetic DMARDs and 110 in the cohort receiving TNFi) (Figure 2). The median follow‐up period per patient was 3.9 years in the synthetic DMARD**–**treated cohort and 5.6 years in the TNFi‐treated cohort. To obtain the approximate half‐way point of the duration of follow‐up, the time periods and events were divided into 2 periods: the first period consisted of the start of follow‐up until the end of the second year (18 ischemic strokes in the synthetic DMARD**–**treated cohort and 49 ischemic strokes in the TNFi‐treated cohort), and the second period consisted of the third year until the end of follow‐up for this data set (6 ischemic strokes in the synthetic DMARD**–**treated cohort and 61 ischemic strokes in the TNFi‐treated cohort). In the TNFi‐treated cohort, the risk of ischemic stroke appeared to be higher in the first period (HR 1.07 [95% CI 0.55**–**2.07]) than in the second period (HR 0.74 [95% CI 0.26**–**2.09]), but the confidence intervals were wide and overlapped. A test for an interaction of the risk over time yielded nonsignificant results (*P* = 0.81).

### All‐cause mortality after first ischemic stroke

Thirteen deaths were reported to have occurred within 30 days of the 127 first ischemic strokes (3 in the patients receiving synthetic DMARDs, 2 in the patients receiving TNFi, and 8 in those who had past exposure to TNFi at the time of the ischemic stroke) (Table 3). Of these patients, 9 (69%) had diseases of the circulatory system (ICD‐10 chapter I) listed as the underlying cause of death, including 4 who died of cerebrovascular disease (I60‐69).

**Table 3:** All‐cause mortality after ischemic stroke in patients receiving synthetic DMARDs, patients receiving TNFi at the time of ischemic stroke, and patients with past exposure to TNFi^a^

	Patients receiving synthetic DMARDs at time of first ischemic stroke (n = 21)	Patients receiving TNFi at time of first ischemic stroke (n = 88)	Patients with past exposure to TNFi at time of first ischemic stroke (n = 18)
All‐cause mortality within 30 days of ischemic stroke			
Number of deaths	3	2	8
Unadjusted OR (95% CI)	Referent	0.16 (0.02–1.04)	2.18 (0.50–9.44)
OR adjusted for age and sex (95% CI)	Referent	0.18 (0.03–1.21)	2.58 (0.57–11.72)
All‐cause mortality within 1 year of ischemic stroke			
Number of deaths	4	8	9
Unadjusted OR (95% CI)	Referent	0.50 (0.13–1.86)	1.82 (0.48–6.96)
OR adjusted for age and sex (95% CI)	Referent	0.60 (0.16–2.28)	2.32 (0.58–9.35)

a DMARDs = disease‐modifying antirheumatic drugs; TNFi = tumor necrosis factor inhibitors; OR = odds ratio; 95% CI = 95% confidence interval.

Compared to the synthetic DMARD–treated cohort, the unadjusted OR of death within 30 days after ischemic stroke in patients receiving TNFi at the time of ischemic stroke was 0.16 (95% CI 0.02–1.04) and in patients with past exposure to TNFi was 2.18 (95% CI 0.50–9.44) (Table 3). After adjusting for age and sex, the adjusted OR was 0.18 (95% CI 0.03–1.21) in the patients who were receiving TNFi at the time of ischemic stroke and 2.58 (95% CI 0.57–11.72) in those with past exposure to TNFi.

By 1 year after ischemic stroke, there were 21 deaths: 4 in the synthetic DMARD–treated group, 8 in the TNFi‐treated group, and 9 in the group of patients who had past exposure to TNFi at the time of ischemic stroke (Table 3). Of the 21 patients who died within 1 year, 12 (57%) had diseases of the circulatory system (ICD‐10 chapter I) listed as the underlying cause of death; of these 12, 5 had cerebrovascular disease (I60‐69) listed as the underlying cause of death. There was a nonsignificant reduction in mortality in patients receiving TNFi at the time of ischemic stroke compared to those receiving synthetic DMARDs (OR 0.60 [95% CI 0.16–2.28]) and a nonsignificant increase among those with past exposure to TNFi (OR 2.32 [95% CI 0.58–9.35]). In all cases, the total number of deaths was very low, with resulting imprecision of these estimates.

## DISCUSSION

The findings of this study indicate that TNFi do not influence the occurrence of ischemic stroke compared to synthetic DMARDs alone in patients with RA over a period of 4–6 years, which is consistent with the findings of previous studies focusing on the risk over the short term 5, 12, 20. Although numbers were small, there was a trend toward a reduction in mortality at 30 days and at 1 year following the event among patients who were actively receiving TNFi at the time of their stroke compared to those who had never received biologic therapies.

Control of inflammation through blockade of TNF and, in turn, atherosclerosis may lead to a reduction in the risk of ischemic stroke. However, ischemic stroke could result from large artery atherosclerosis, cardioembolic phenomena, and systemic hypoperfusion. Although atherosclerotic processes likely underlie the first two pathogenic mechanisms, they may not account for systemic cerebral hypoperfusion and TNF may not play a role in this situation. It was not possible to subtype ischemic stroke in the present study, since no data were obtained on carotid Doppler imaging and echocardiograms.

In animal studies of stroke, TNF inhibition via different mechanisms (TNF‐knockout mice or antibodies to TNF or TNF‐binding proteins) has been shown to limit the size of infarct territory (for review, see ref. 11). In humans, infarct size has been correlated with mortality 21, 22. Analysis of 30‐day and 1‐year mortality did not demonstrate any definite influence of TNFi on outcome after ischemic stroke, although the number of deaths was very low. However, the direction of the point estimate differed between patients receiving TNFi at the time of ischemic stroke versus those with past exposure, with the risk of mortality increased in the latter group. The lower mortality rates in the patients actively receiving TNFi may indicate a possible benefit which warrants further study. It is possible that in the prior exposure group, the reason the patient was no longer receiving TNFi (e.g., lack of response, adverse events, and comorbidity) may itself have influenced their risk of death.

The prospective design of the BSRBR‐RA meant it was possible to collect detailed information on drug exposure and events of interest and assess the relationship between the two. The amount of data collected allowed for extensive confounder adjustment compared to previous studies. For example, data on potential confounders such as smoking, hypertension, and hyperlipidemia (statin use) were captured in this study. This study used propensity scores as a method to balance these and other known confounders in order to reduce the risk of model misspecification compared to traditional multivariate analysis. The use of propensity scores to adjust for confounding was successful, as demonstrated by the low levels of expected bias (<5%) obtained using stratification by deciles of propensity score.

AF is an important risk factor in the pathophysiology of ischemic stroke, but baseline data on the presence or absence of AF was not collected in the BSRBR‐RA. Therefore, digoxin and/or warfarin use was a proxy for AF. Patients receiving these drugs were grouped together, since the numbers were low in each drug group. Warfarin alone can also be used as an anticoagulant for other conditions, such as deep vein thrombosis. Since the indication for warfarin was not always clear from the clinical baseline questionnaire, a decision was made that all patients receiving digoxin and/or warfarin were assigned the AF proxy, allowing for possible misclassification as a limitation.

The WHO criteria were used to verify strokes, but these criteria do not differentiate between the ischemic and hemorrhagic subtypes. Particular attention was paid to subtyping these strokes using CT brain reports for physician‐reported strokes and ICD‐10 codes for strokes reported via death certificates. Using brain imaging reports, the majority of strokes subtyped in this analysis were ischemic strokes, consistent with general stroke epidemiology 23, 24. This method of using brain CT reports to verify stroke subtype has been described previously 25. For example, it was used in a population‐based cohort study to evaluate the accuracy of identifying incident stroke by linkage with hospital records and the UK national death register 26. A high positive predictive value of 84% was obtained, suggesting that this is a valid approach. In this study, a sensitivity analysis showed no difference in stroke subtype between treatment cohorts. This suggests that the association with TNFi did not differ between stroke subtypes and that physicians were not preferentially reporting one stroke subtype over another in either treatment cohort.

It is possible that there could be differential reporting of strokes in general to the register if physicians treating patients with TNFi reported more strokes because of perceived safety concerns as opposed to synthetic DMARD therapy. At present, there is no mechanism to ascertain whether there is differential underreporting of strokes to the register. Of the 259 strokes reported to the register, there was insufficient clinical information to verify the event for 37 (14%), but it was unlikely that clinicians preferentially supplied additional clinical data for the TNFi‐treated patients compared to the synthetic DMARD–treated patients, since the proportion of verified strokes (181 of 222 [82%]) was similar to the proportion of unverified strokes (30 of 37 [81%]) in the TNFi‐treated patients (Figure 2).

The use of ICD‐10 codes to verify strokes reported via death certificates could be perceived as a limitation, but since death certificates are completed by a physician, it is likely that there were supporting clinical data leading up to death to make the diagnosis of stroke. Therefore, the events included in the analysis represent definite events and the crude incidence rates reported reflect the minimum event rates.

Despite the large size of the BSRBR‐RA, the actual number of strokes was low. With the number of patients and length of follow‐up time currently available, the power to detect a doubling in the risk of ischemic stroke was 91%, and the power to detect a halving in risk was 69%. In order to be able to detect a 10% reduction in risk with 80% power, one would need much longer follow‐up or increased patient recruitment. An alternative would be to combine patients from different observational studies to increase patient numbers for analysis.

The ever‐exposed drug model was chosen for the primary analysis because it was hypothesized that blockade of TNF likely influences all stages of atherosclerosis progression and subsequent development of cardiovascular events over the medium to long term. Exploration of other drug exposure models was done as sensitivity analyses. Although the point estimates of the individual drug models differed, the 95% CIs overlapped, which suggests that it is likely due to low statistical power. Replication in other data sets is warranted.

In summary, this study indicates that TNFi therapy is not associated with the occurrence of ischemic stroke in patients with RA over the medium term within this large national RA biologics register.

## AUTHOR CONTRIBUTIONS

All authors were involved in drafting the article or revising it critically for important intellectual content, and all authors approved the final version to be published. Dr. Hyrich had full access to all of the data in the study and takes responsibility for the integrity of the data and the accuracy of the data analysis.

**Study conception and design.** Low, Lunt, Mercer, Watson, Dixon, Symmons, Hyrich.

**Acquisition of data.** Low, Lunt, Mercer, Watson, Dixon, Symmons, Hyrich.

**Analysis and interpretation of data.** Low, Lunt, Mercer, Watson, Dixon, Symmons, Hyrich.

## Supporting information


**Supplementary Figure 1.** Expected bias of each confounder before construction of PS and after stratification by deciles of PS.

**Supplementary Table 1.** Balance of distribution of baseline confounders between sDMARD and TNFi patients before creating the PS model and after stratification by deciles of PS.

**Supplementary Methods**
