# Supplementary material for: Association Between Ischemic Stroke and Tumor Necrosis Factor Inhibitor Therapy in Patients With Rheumatoid Arthritis
Source: Arthritis Rheumatol. 2016 May 26;68(6):1337–45. doi: 10.1002/art.39582 (PMC4982051; doi:10.1002/art.39582)
Supplement: Supplementary file 1 — Supplementary Figure 1. Expected bias of each confounder before construction of PS and after stratification by deciles of PS. [file ART-68-1337-s002.doc]

**Supplementary Figure 1.** Expected bias of each confounder before construction of PS and after stratification by deciles of PS.
