# Supplementary material for: Association Between Ischemic Stroke and Tumor Necrosis Factor Inhibitor Therapy in Patients With Rheumatoid Arthritis
Source: Arthritis Rheumatol. 2016 May 26;68(6):1337–45. doi: 10.1002/art.39582 (PMC4982051; doi:10.1002/art.39582)
Supplement: Supplementary file 2 — Supplementary Table 1. Balance of distribution of baseline confounders between sDMARD and TNFi patients before creating the PS model and after stratification by deciles of PS. [file ART-68-1337-s003.doc]

**Supplementary Table 1.** Balance of distribution of baseline confounders between sDMARD and TNFi patients before creating the PS model and after stratification by deciles of PS.

| **Variable** | **Before creating PS** | | | **Stratification by deciles of PS** | | |
| --- | --- | --- | --- | --- | --- | --- |
|  | **sDMARD** | **TNFi** | **% Expected bias** | **sDMARD** | **TNFi** | **% Expected bias** |
| **Demographics** |  |  |  |  |  |  |
| Mean age, years | 59.9 | 56.0 | -19.2 | 56.1 | 56.0 | -1.0 |
| Female gender, % | 73.5 | 76.5 | -1.8 | 76 | 77 | -0.1 |
| **Disease-related factors** |  |  |  |  |  |  |
| Median disease duration, years | 9.8 | 13.2 | -0.8 | 13.1 | 13.2 | 0 |
| Mean DAS28 | 5.3 | 6.6 | -15.5 | 6.5 | 6.6 | -0.8 |
| Mean HAQ | 1.5 | 2.0 | 26.3 | 2.0 | 2.0 | 1.1 |
| Study entry prior to 2004, % | 19.2 | 51.7 | 21.0 | 51 | 52 | 0.2 |
| Use of >4 sDMARDs, % | 21.3 | 52.3 | 10.6 | 52 | 52 | 0.2 |
| **Cardiovascular risk factors** |  |  |  |  |  |  |
| Hypertension, % | 31.6 | 29.9 | -0.2 | 30 | 30 | 0 |
| Diabetes,% | 6.6 | 5.6 | 0.1 | 6 | 6 | 0 |
| Chronic lung disease, % | 19.2 | 13.6 | -1.6 | 14 | 14 | -0.1 |
| Ever smoker, % | 63.3 | 59.8 | -1.0 | 60 | 60 | 0.1 |
| **Drugs** |  |  |  |  |  |  |
| Glucocorticoid, % | 22.4 | 44.2 | 6.2 | 44 | 44 | 0.1 |
| NSAID/COX, % | 55.3 | 62.7 | -1.6 | 63 | 63 | 0 |
| Antiplatelet, % | 11.3 | 6.8 | -1.2 | 7.0 | 7.0 | 0 |
| Statin, % | 12.7 | 7.1 | 1.8 | 7.0 | 7.0 | 0 |
| Digoxin and/or warfarin, % | 2.0 | 1.7 | 0.6 | 2.0 | 2.0 | 0 |
