# Supplementary material for: Association Between Ischemic Stroke and Tumor Necrosis Factor Inhibitor Therapy in Patients With Rheumatoid Arthritis
Source: Arthritis Rheumatol. 2016 May 26;68(6):1337–45. doi: 10.1002/art.39582 (PMC4982051; doi:10.1002/art.39582)
Supplement: Supplementary file 3 — Supplementary Methods [file ART-68-1337-s001.doc]

## Methods

### Expected bias

The expected bias is defined as the difference in the outcome due to the imbalance between each confounder, taking into account the strength of the relationship between each confounder and outcome. Using a matrix of the beta coefficients of the estimated effect of each covariate on the outcome, the degree of expected bias is calculated. This value is the sum of the beta coefficients, taking into account the direction of the effect, some of which were in a negative direction, decreasing the estimate and some of which were in a positive direction, increasing the estimate, thus cancelling each other out. If the direction of the individual coefficients were ignored, one would then obtain the absolute value of the expected bias. This is a better measure of the degree of bias in the analysis compared to using the beta coefficients as it shows how different the two cohorts are from each other and the degree of bias in the estimated treatment effect. A maximum bias of 5% in either direction was considered to be an acceptable threshold. Before the propensity score (PS) was created, the absolute degree of bias that would have resulted from the confounders was very high: 192.3%. Patients were then stratified by deciles of PS, resulting in the least amount of bias (3.8%) hence deciles of PS was used in the final regression model for confounder adjustment. Additional sensitivity analyses were also undertaken with trimming of the propensity score at 1%, 5% and by exclusion of the most extreme deciles. The expected bias of each confounder before and after stratification by deciles of PS is shown in Supplementary Figure 1 and tabulated in Supplementary Table 1.

### Missing data

Missing data were imputed for the following covariates: DAS28 score, disease duration, smoking status and HAQ. This was performed using the ice command in Stata. Imputation was performed separately for TNFi and sDMARD cohorts, with 20 cycles of imputations each, resulting in 20 unique datasets. Each dataset was analysed using standard regression modelling and the final estimate combined using the mim command (according to Rubin’s rules) in Stata. Stata version 12 was used (StataCorp, College Station, Texas).

The proportion of missing baseline data for the baseline covariates was <1% for both sDMARD and TNFi cohorts with the exception of HAQ score. There was a high proportion of missing data for this variable; 20% in the sDMARD cohort and 5% in the TNFi cohort.
